# The Dickkopf-1 (DKK1) Dichotomy in Oncology: New Insights on Tumor Progression and Immune Regulation

**DOI:** 10.3390/ijms27093780

**Published:** 2026-04-23

**Authors:** Alessandro Canella, Zachary Gentry, Casey Cosgrove

**Affiliations:** Department of Obstetrics and Gynecology, Division of Gynecologic Oncology, The Ohio State University Comprehensive Cancer Center, James Cancer Hospital, Columbus, OH 43210, USA

**Keywords:** DKK1, Dickkopf-1, Wnt, CKAP4, immunosuppression, immunoregulation, immunotherapy, biomarker, oncogene, metastasis

## Abstract

Dickkopf-1 (DKK1) is a 266-amino-acid secreted glycoprotein originally identified as a high-affinity antagonist of the canonical Wnt/β-catenin signaling pathway and has emerged as a complex regulator in oncology. While historically considered as a tumor suppressor due to its ability to abrogate Wnt-driven proliferation, recent discoveries highlight a paradoxical pro-oncogenic role across various malignancies. The molecular mechanisms by which DKK1 promotes tumor progression, metastasis, and immune evasion are driven by its interaction with cell-surface receptors, specifically LRP5/6 and CKAP4. The DKK1-CKAP4 axis independently activates PI3K/AKT signaling, facilitating epithelial–mesenchymal transition (EMT), chemoresistance, and the formation of osteolytic bone lesions. Furthermore, DKK1 serves as a critical orchestrator of the tumor microenvironment (TME) by driving comprehensive immune reprogramming. It mediates the recruitment of myeloid-derived suppressor cells (MDSCs) and inactivates cytotoxic CD8^+^ T cells and natural killer (NK) cells, thereby fostering an immunosuppressive tumor microenvironment and resistance to checkpoint inhibitors. Interestingly, cancer-associated fibroblasts (CAFs) are a primary source of DKK1 in the stroma, where they facilitate immune evasion. Clinically, elevated circulating DKK1 levels correlate with advanced disease stages, increased metastatic potential, and poor overall survival in solid and hematological tumors. When used in combination with established biomarkers, serum DKK1 levels demonstrate significant utility for early detection and therapeutic monitoring. Given its intricate impact on malignancy, DKK1 has become a promising therapeutic target, with ongoing clinical trials investigating neutralizing antibodies such as DKN-01 to disrupt its oncogenic and immunosuppressive signaling. Understanding the context-dependent nature of DKK1 signaling remains essential for refining its application as both a biomarker and a component of emerging precision immunotherapy strategies. By prioritizing the literature from the last decade, this review characterizes DKK1 as a key mediator of tumor progression and immune reprogramming, while assessing its clinical potential as a biomarker and therapeutic target.

## 1. Introduction

Dickkopf-1 (DKK1) is a secreted glycoprotein, and a high-affinity natural antagonist of the canonical Wnt/β-catenin signaling pathway. As the most comprehensively studied member of the DKK1 family (DKK1, DKK2, DKK3, and DKK4), DKK1 has been extensively investigated due to its significant roles in osteogenesis [1,2], neurodegenerative diseases [3,4,5], and oncology [6,7,8]. The discovery of DKK1 and its inhibitory role in *Xenopus* embryos was reported for the first time in 1998 [9]. In this study, the Wnt pathway inhibition mediated by DKK1 caused oversized head structures during embryonic development; therefore, the authors named the protein “Dickkopf”, a German term meaning “thick (or fat) head”.

While its role as a Wnt antagonist is well documented in developmental biology, DKK1 also contributes to heart muscle development [10]. Furthermore, it is a critical regulator of hematopoietic regeneration following total body irradiation, where it suppresses mitochondrial senescence and accelerates hematopoietic reconstitution [11]. Beyond these regulatory functions, DKK1 overexpression is known to adversely affect the maintenance of bone homeostasis [12].

Assessing the function of DKK1 in adult cancer physiology and pathology remains complex and challenging. Although DKK1 was historically classified as a tumor suppressor based on its ability to abrogate Wnt-driven cancer cell proliferation, recent evidence highlights its dual role as a pro-oncogenic factor. By suppressing the canonical Wnt pathway or interacting with the cytoskeleton-associated protein 4 (CKAP4) receptor, DKK1 can promote both tumor progression and immunosuppression. Furthermore, elevated serum levels of DKK1 have been identified as an independent poor prognostic biomarker in various solid tumors, including multiple myeloma, esophageal squamous cell carcinoma, breast, cervical, and pancreatic cancers, and are associated with increased metastasis and resistance to chemotherapy and immunotherapies [13,14,15,16,17]. Notably, over the last decade, DKK1 has emerged as a key modulator of the tumor microenvironment (TME), involved in the reprogramming of cancer-associated fibroblasts, cytotoxic T and NK cells, and immunosuppressive myeloid cells.

This review summarizes the most recent and relevant findings regarding the role of DKK1 in cancer, with a primary focus on its involvement in shaping the tumor microenvironment and in promoting tumor progression, metastasis, immune evasion, and drug resistance. Additionally, we discuss its emerging potential as a therapeutic target across multiple tumors.

## 2. Dickkopf-1 (DKK1)

### 2.1. Gene, Protein Structure, and Biological Properties

The human *DKK1* gene locus is on chromosome 10q24.2. It consists of four exons, which are highly conserved across species [17,18]. Transcription is regulated by several transcription factors, including the tumor suppressor p53, which induces DKK1 expression in response to DNA damage, and the Wnt pathway itself, through a negative feedback loop by stimulating DKK1 production via TCF/LEF transcription factors [19].

The *DKK1* mRNA transcript is approximately 1.844 kb in length. Translation yields an initial polypeptide precursor of 266-amino-acid, including a 31-amino-acid N-terminal signal peptide that directs the protein to the secretory pathway [20]. While no alternative *DKK1* splice isoforms have been experimentally detected and reported in the literature, the in silico prediction of a regulatory intronic variant (rs1569198) generating a new splice acceptor site in intron 3 suggests that *DKK1* transcript processing may be subject to context-dependent modulation [21].

**Figure 1:**
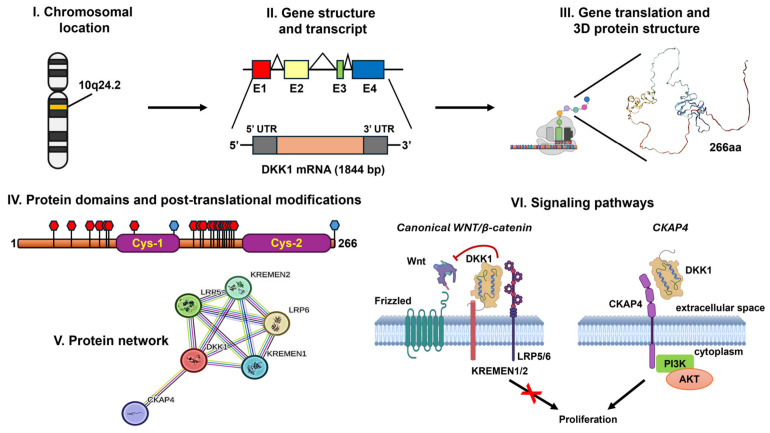
**Summary of the molecular features of DKK1**. (**I**) Chromosomal localization of the *DKK1* gene. (**II**) Gene transcription and mRNA processing (E = exon). (**III**) Protein translation and predicted 3D conformational folding. (**IV**) Linear protein structure highlighting the main cysteine-enriched domains (Cys-1/Cys-2, **purple box**) and post-translational modifications, including O-glycosylation (**red**) and N-glycosylation (**blue**) sites. (**V**) The human DKK1 protein interaction network (string-db.org). (**VI**) Canonical (WNT/β-catenin) and alternative (CKAP4-AKT) signaling pathways modulated by DKK1. Figure created in part with BioRender.com (https://app.biorender.com/illustrations/69711fc61aa57d431ef9eb27, accessed on 16 April 2026).

Following signal sequence cleavage and post-translational modifications, the mature DKK1 protein is secreted into the extracellular space (Figure 1I,II). DKK1 is a secreted glycoprotein that serves as a high-affinity antagonist of the canonical Wnt/β-catenin signaling pathway [17,20]. Structurally, DKK1 is characterized by two distinct and highly conserved cysteine-rich domains (CRDs), known as the N-terminal (DKK1N) and C-terminal (DKK1C) domains, which are separated by a flexible, less-conserved linker region [20,22] (Figure 1III).

The DKK1N domain contains five unique intramolecular disulfide bonds. These bonds arrange in a topology specific to the DKK family, with no direct structural matches in other known disulfide clusters [20]. In contrast, the DKK1C domain, while also containing five intramolecular disulfide bonds, exhibits significant structural homology with the disulfide-rich fold colipase and the “cysteine knot” family [20,23]. These domains are critical for functional versatility, mediating high-affinity interactions with a variety of cell-surface receptors [22,24] (Figure 1IV).

The molecular weight of human DKK1 (huDKK1) is characterized by significant heterogeneity due to extensive post-translational modifications, primarily glycosylation. While the predicted molecular weight based on its amino-acid sequence is approximately 25.8 kDa, experimental observations via SDS-PAGE and MALDI-MS typically reveal distinct forms at approximately 27.4 and 29.5 kDa [20]. This shift is primarily driven by both N- and O-linked glycosylation [19,20]. Up to 21 glycosylation sites have been described in the literature for DKK1 (Figure 1IV): O-glycosylation occurs at multiple sites within the polypeptide backbone, at Ser30, Asn44, Ser 61, Tyr68, Tyr74, Thr76, Ser99, Gly148, Thr153, Thr155, Ser163, Thr164, Tyr168, Ser169, Thr172, Thr173, Ser175, Met178, Tyr179, Thr181 [20,25,26]; N-glycosylation predominantly occurs at Asn131, Asn266, Asn225 [20,26,27,28]. Notably, approximately 92% of huDKK1 molecules undergo N-linked glycosylation at Asn225 [20,26]. The most prevalent structures identified include GalNAc (sialic acid)-Gal-sialic acid (comprising 65% of the total) and GalNAc-Gal [sialic acid] (30%). These N-glycans are structurally diverse, including biantennary forms with one or two sialic acids (23% and 60%, respectively) and a triantennary form with two sialic acids (9%) [20] (Figure 1IV). These modifications are not merely aesthetic. They are essential for the protein’s stability, protection against proteolysis, secretion efficiency, and proper folding within the complex extracellular environment [19].

The biological function of DKK1 is primarily driven by its high-affinity interactions with several transmembrane receptors. DKK1 antagonizes canonical Wnt signaling by binding with high affinity to the extracellular domains of the LRP5/6 co-receptors [20,23]. Crystallographic analyses demonstrate that the DKK1C domain is the primary driver of this interaction. Specifically, DKK1C binds to the top surface of the LRP6-E3 YWTD β-propeller domain [22,23]. This interaction involves conserved hydrophobic regions on DKK1C, specifically residues Phe225 and Trp232, which insert into the central cavity of the LRP6 propeller, further stabilized by a peripheral network of salt bridges and hydrogen bonds [23,29]. Due to the structural similarity between the E1 and E3 β-propellers, DKK1C is also believed to interact with the LRP6-E1 domain [22]. By occupying these sites, DKK1 competitively displaces Wnt ligands (such as Wnt3a) from binding, preventing the assembly of the Wnt-Frizzled-LRP complex and subsequent downstream signaling [22,29].

To further suppress signaling, DKK1 recruits the transmembrane receptors Kremen-1 and Kremen-2 [17,24]. This binding facilitates the formation of a ternary DKK1-LRP6-Kremen complex, which triggers rapid internalization and endocytosis of LRP5/6 [29,30]. This process effectively depletes the co-receptors from the plasma membrane, leading to sustained and potent inhibition of the Wnt pathway [17] (Figure 1V,VI).

Recent studies have identified CKAP4 as an alternative high-affinity receptor for DKK1, with a dissociation constant (Kd) of approximately 0.5 nM [24]. The protein domains involved in the binding are CRD1 for DKK1, and leucine zipper (LZ) for CKAP4 [31,32,33]. Unlike its inhibitory role in Wnt signaling, the DKK1-CKAP4 axis activates the PI3K/AKT signaling pathway, promoting tumor-cell proliferation and metastasis independently of the canonical Wnt/β-catenin pathway [19,24,31,32,34,35]. This multi-receptor dual affinity underscores the structural complexity and context-dependent role of DKK1 as either a signaling inhibitor or an oncogenic driver [24] (Figure 1V,VI).

### 2.2. DKK1 in Cancer: The Signaling Paradox

**Figure 2:**
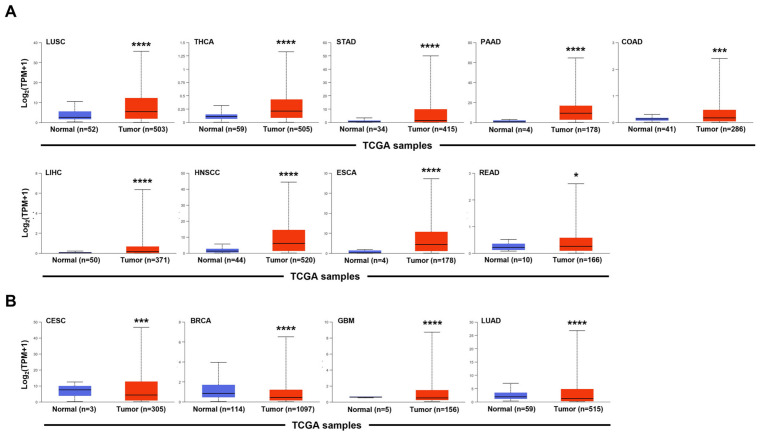
Transcriptomic profiling of DKK1 expression, quantified in transcripts per million, across TCGA cohorts. (Primary malignant tissues in red, matched or adjacent normal control tissues in blue; ualcan.path.uab.edu) [37]. (**A**) Significant transcriptional upregulation was observed in colon adenocarcinoma (COAD), esophageal carcinoma (ESCA), head and neck squamous cell carcinoma (HNSCC), liver hepatocellular carcinoma (LIHC), lung squamous cell carcinoma (LUSC), pancreatic adenocarcinoma (PAAD), rectum adenocarcinoma (READ), stomach adenocarcinoma (STAD), and thyroid carcinoma (THCA). (**B**) Significant transcriptional downregulation was observed in cervical squamous cell carcinoma (CESC), breast invasive carcinoma (BRCA), glioblastoma (GBM), and lung adenocarcinoma (LUAD). Gene expressions are expressed in logarithmic scale. TPM: transcripts per million. Unpaired two-sample *t*-tests were used to evaluate significance in expression levels (* *p* < 0.05, *** *p* < 0.005, **** *p* < 0.0001). Sampling biases and platform-specific effects, due to uneven distribution of normal controls across cohorts, may influence the statistical significance of normal–tumor comparisons.

**Figure 3:**
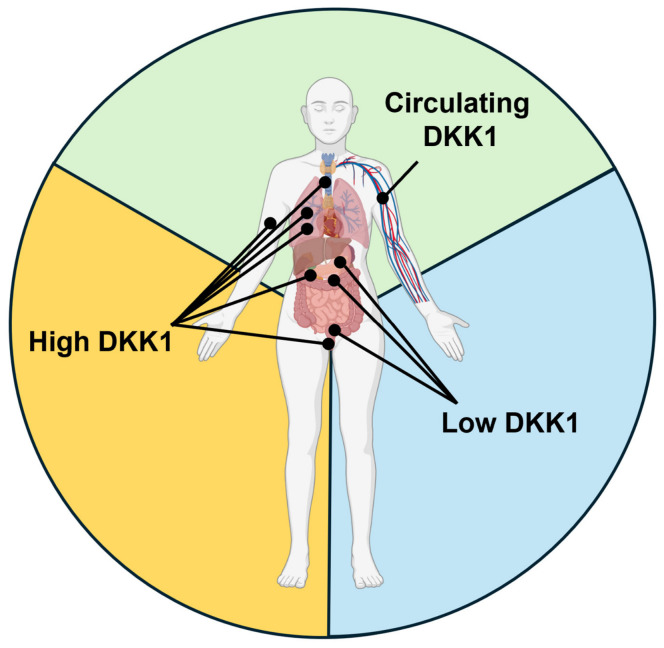
**Mapping the expression and distribution of DKK1 across human physiology and oncology.** The figure illustrates the systemic distribution and relative expression profiles of DKK1 in the human body. The diagram is divided into three key physiological compartments: circulating DKK1 (green sector), tumors characterized by oncogenic DKK1 overexpression (yellow sector), and tumors associated with DKK1 downregulation (blue sector). Figure created in part with BioRender.com (https://app.biorender.com/illustrations/697cf895a8e30ffe71e972aa, accessed on 16 April 2026).

Most tumors exhibit aberrant activation of the canonical Wnt signaling pathway [36]. Although inhibiting the LRP5/6-DKK1 interaction should theoretically suppress cancer cell proliferation and tumor growth, DKK1 is paradoxically overexpressed in many invasive cancers (Figure 2A). In these tumors, DKK1 is actively secreted into the TME, where it functions as a potent signaling hub, and is released into the bloodstream to promote metastasis. Transcriptomic profiling based on The Cancer Genome Atlas (TCGA) reveals that compared to normal tissues, DKK1 expression is significantly upregulated in patients affected by a number of cancers, including colon adenocarcinoma, esophageal carcinoma, head and neck squamous cell carcinoma, hepatocellular carcinoma, lung squamous cell carcinoma, pancreatic adenocarcinoma, rectum adenocarcinoma, stomach adenocarcinoma, and thyroid carcinoma (Figure 2A). In contrast, tumors with significantly lower relative expression of DKK1 compared to normal tissue include squamous cell cervical carcinoma, invasive breast carcinoma, and lung adenocarcinoma (Figure 2B). Beyond its traditional role as a Wnt pathway antagonist, elevated extracellular DKK1 promotes tumor progression through the activation of the CKAP4/PI3K/Akt pathway, facilitating metastasis, osteolytic bone lesions, and immunosuppression. These mechanisms allow malignant cells to bypass both metabolic checkpoints and immune surveillance. Therefore, certain tumors benefit from either high extracellular DKK1 levels or reduced DKK1 expression in the TME, or increased DKK1 circulation in the bloodstream to drive disease progression (Figure 3).

#### 2.2.1. Role in Tumor Progression, Metastasis, and Chemoresistance

The first pioneering publication establishing the crucial role of DKK1 in cancer was published over two decades ago, in the context of hematological malignancies. This landmark study demonstrated that DKK1 is secreted by multiple myeloma (MM) cells and that systemically elevated circulating levels impair osteoblast differentiation by blocking canonical Wnt signaling. This disruption directly mediated the pathogenesis of osteolytic bone lesions and promotes bone destruction in patients [6].

At that time, the study did not address the molecular mechanisms responsible for DKK1 upregulation in myeloma cells. In 2021, a study uncovered a hypoxia-dependent regulatory mechanism controlling DKK1 gene expression. Under hypoxic conditions, activation of the p38 MAPK signaling pathway induces phosphorylation and nuclear translocation of CREB. In the nucleus of multiple myeloma cells, CREB cooperates with the histone methyltransferase MMSET to enrich H3K36me2 occupancy at the *DKK1* promoter, triggering transcriptional activation. Of note, the authors also demonstrated that the combination of CREB inhibition and treatment with the hypoxia-activated prodrug TH-302 (DNA-alkylating agent) significantly reduced bone lesions in a multiple myeloma murine model [38]. Conversely, epigenetic regulation via miR-342 and miR-363 can suppress *Runx2* expression and its downstream targets *RANKL* and *DKK1*, thereby inhibiting the expression of markers associated with MM cell invasion and migration in vitro and in vivo [39]. Beyond its suppressive effect on osteoblasts, DKK1 functions as an autocrine/paracrine ligand for the CKAP4 receptor in multiple myeloma cells. This interaction enhances the activation of the non-canonical NF-kB pathway, conferring resistance to the proteasome inhibitor bortezomib in vivo [35]. Over the past decade, several studies have expanded the knowledge of the role and clinical significance of DKK1 in solid tumors, extending its oncological relevance beyond hematological malignancies (Figure 3).

As outlined in the previous section, TCGA data reveal significant and aberrant upregulation of DKK1 in carcinomas, adenocarcinomas, and germ cell tumors compared to normal tissues [40,41]. DKK1 is a robust prognostic factor in breast cancer (BC) [42], and novel insights have increased knowledge of the mechanisms of DKK1 gene regulation. Sox2 and Sox9 are directly involved in driving the autocrine expression of DKK1, facilitating immune evasion from natural killer (NK) cells and metastatic latency until immune surveillance is reactivated [43]. Similar to the findings in MM [38], p38 MAPK was reported as an upstream regulator of *DKK1* expression in breast cancer, suggesting that p38 inhibition could indirectly mitigate DKK1-driven bone disruption [44]. Conversely, DKK1 exhibits context-dependent suppressive roles in specific BC models. In 2019, Niu et al. [45] demonstrated in vitro that DKK1 is responsible for the inhibition of cell migration and invasion by abrogating β-catenin-dependent MMP7 transcription, thereby limiting extracellular matrix degradation and tumor invasion [46,47]. Additionally, another study identified PRMT5 as an epigenetic promoter of canonical Wnt/β-catenin signaling by transcriptionally silencing DKK1 and DKK3. The epigenetic mechanism involves PRMT5-mediated histone methylation at the *DKK1* and *DKK3* promoters, resulting in gene silencing and subsequent increase in cellular growth, migration, and invasion, particularly in aggressive triple-negative breast cancer (TNBC) [48]. DKK1 can be upregulated in post-chemotherapy cancer cells, contributing to clinical morbidity. In vivo treatment of breast cancer with paclitaxel upregulates the EGFR signaling pathway, enhancing DKK1 upregulation. In addition, DKK1 inhibits the Wnt-PCP-JNK pathway and *PTGS2* expression in breast cancer SCP28 cells, thereby promoting migration, lung metastasis, and immunosuppression [49]. Beyond the effect on breast cancer cells, paclitaxel-induced EGFR signaling triggers secondary upregulation of DKK1 within dorsal root ganglion neurons, causing chemotherapy-induced peripheral neuropathy [50].

In prostate cancer (PC), promoter hypomethylation drives DKK1 overexpression, though this epigenetic regulatory mechanism appears tumor-specific [51]. In addition, the p38 MAPK signaling pathway has been identified as a regulator of DKK1 expression in PC. Among the p38 isoforms, MAPK11 controls DKK1 secretion across different stages of osteolytic prostate cancer. Conversely, PC cells can also downregulate DKK1 expression in bone metastasis. The parathyroid hormone-related protein (PThrP) is produced by PC cells and is associated with tumor progression and metastasis. During PC progression, PThrP upregulates c-Jun expression, which binds the DKK1 promoter at the Wnt response element (WRE) motif site 2, repressing gene transcription [52]. Despite this discovery, the ability of DKK1 to impair osteoblast differentiation and induce bone lesions remains a hallmark of PC pathology [53]. To further support the role of DKK1 in prostate cancer metastasis and bone disruption, recent evidence further demonstrates that DKK1 enhances cancer cell proliferation, migration, and epithelial–mesenchymal transition (EMT) via JNK-mediated apoptosis and activation of NF-kB signaling. Within the bone microenvironment, DKK1 inhibits osteoblast differentiation, resulting in diminished intramedullary bone formation and enhanced bone destruction [7,54].

In lung cancer, DKK1 expression is associated with vasculogenic mimicry (VM) [55,56], a process wherein tumor cells acquire endothelial-like characteristics to organize into functional tubular structures that supply nutrients and blood to the tumor core. Furthermore, DKK1 promotes EMT and the cancer stem-like cell phenotype, conferring resistance to chemotherapy and radiotherapy. These factors collectively correlate with poor patient survival, identifying DKK1 as a driver of tumor progression in non-small cell lung cancer (NSCLC) [57,58].

Novel insights into the DKK1-CKAP4 signaling pathway have been reported by recent studies in pancreatic and esophageal tumors. In pancreatic cancer, DKK1 has a significant role in tumor progression, and serves as a poor prognostic factor [59]. The DKK1 promoter is targeted by miR-33a-5p. In esophageal cancer patients the silencing of miR-33a-5p leads to DKK1 upregulation, which maintains a partially differentiated, aggressive cellular phenotype associated with poor prognosis [60]. Interestingly, DKK1 overexpression in esophageal adenocarcinoma (EAC) enhances Akt phosphorylation, most likely through the binding of the CKAP4 receptor and independently of the canonical Wnt/β-catenin pathway. Notably, DKK1 silencing significantly reduces EAC cell proliferation, invasion, and survival [61]. In both pancreatic and esophageal tumors, FOXM1 has been identified as a key downstream transcription factor of the DKK1-CKAP4-Akt axis. DKK1-mediated Akt activation synergizes with MEK-ERK signaling to induce *FOXM1* expression and promote cancer cell proliferation. Intriguingly, FOXM1 is directly involved in driving *DKK1* gene transcription, due to a Wnt-independent positive feedback loop that correlates with increased malignancy and poor prognosis [62].

Even though increased DKK1 levels in several tumors are promoters of tumor progression and metastatic programs, defining its biological role in gastric cancer (GC) seems more challenging and clinically controversial. While elevated DKK1 levels typically correlate with advanced high N-stage and poorer survival [63], high DKK1 expression in stage IIB–IIIC patients treated with XELOX (oxaliplatin and capecitabine) has been identified as a strong predictor of better 5-year overall survival (OS) [64]. In this tumor, several regulatory mechanisms of DKK1 transcriptional suppression are affecting DKK1. *FOXC1* is highly expressed in GC patients and correlates with poor prognosis. Jiang J et al. demonstrated that FOXC1 binds the *DKK1* promoter at two distinct sites, causing *DKK1* silencing. The consequence is the promotion of cancer cell proliferation through the upregulation of the Wnt/β-catenin pathway [65]. Overexpression of miR-501-5p causes silencing of *DKK1*, *NKD1*, and *GSK3β*, leading to aberrant upregulation of the Wnt/β-catenin pathway, enhanced cancer stemness phenotype (CD133, Bmi1, Nanog, MYC, SOX2), and tumor progression [66]. In another study, the authors showed that miR-493 is responsible for *DKK1* silencing in GC cells, hence enhancing proliferation, and conferring chemoresistance to cisplatin (cDDP). Although miR-493 correlates with TNM (Tumor–Nodes–Metastasis) staging and distant and lymph node metastasis, the overexpression of DKK1 subverts the tumor progression effect mediated by miR-493 in GC in vivo [67]. Among the regulatory mechanisms of *DKK1* gene expression by non-coding RNA, long non-coding RNAs (lncRNAs) are directly involved. The lncRNA UC.145 is a potential prognostic marker in GC and is involved in cancer cell proliferation. Interestingly, UC.145 targets the lncRNA PRKG1-AS, boosting the expression of *EZH2*, a histone methyltransferase and one of the master epigenetic regulators of gene transcription. EZH2 methylates the *DKK1* promoter, leading to transcriptional repression and subsequent upregulation of the Wnt pathway to support tumor progression [68]. Despite these suppressive mechanisms, the relationship between DKK1 and cancer progression appears well characterized in GC tumors. However, one study reports opposite results: DKK1 is upregulated in cisplatin-resistant GC cell lines, where it orchestrates chemoresistance to cisplatin (cDDP) and EMT through PI3K-Akt pathway activation [69].

In head and neck squamous cell carcinoma (HNSCC) patients, high intratumoral DKK1 expression correlates with the upregulation of wound-healing and angiogenic biological processes. Compared to HNSCC patients with low intratumoral DKK1, this molecular profile drives radioresistance and is significantly associated with poorer clinical survival [8].

In colorectal cancer (CRC), an indirect regulatory mechanism of DKK1 expression has been recently characterized. FGR, a Src family non-receptor tyrosine kinase, induces DKK1 expression through the PI3K-Akt pathway, which facilitates the recruitment of the transcription factor SP1 to the *DKK1* promoter, driving transcriptional upregulation in cancer cells [70]. The prognostic value of DKK1 in CRC appears highly context-dependent.

High levels of S100A4 combined with low DKK1 protein expression levels are associated with the worst five-year survival rate. Conversely, the co-expression of S100A4 and DKK1 is a favorable prognostic factor in CRC, suggesting that DKK1 may partially attenuate the aggressive S100A4-associated phenotype [71]. Despite these protective associations, DKK1 is significantly upregulated in relapsed CRC patients following oxaliplatin therapy (DNA-intercalating agent). In these patients, the upregulation of DKK1-CKAP4-Akt signaling has been recognized as a mechanism of acquired chemoresistance to oxaliplatin [33]. Epigenetic silencing through promoter hypermethylation of the DKK1 promoter is very common in colorectal cancer with microsatellite instability (MSI) due to hypermethylated *MLH1*, *BRAF V600E* mutation, and proximal tumor localization [72]. *DKK1* hypermethylation at the 5′-UTR, and consequently reduced DKK1 in serum and tissues, correlates with perineural invasion and vitamin D deficiency in CRC patients. Notably, vitamin D-mediated and locus-specific CpG demethylation triggers the epigenetic reactivation of *DKK1* expression, which significantly affects CRC cell proliferation and migration [73]. Interestingly, recent evidence proposed a novel, non-canonical nuclear function of DKK1 in CRC. Based on the authors’ findings, and independently of Wnt/β-catenin signaling, DKK1 can localize to regions of open chromatin. In this way, DKK1 regulates the overexpression of *ALDH1A1* and *REPS2*, thus conferring chemoresistance and significantly reducing overall survival (OS) and progression-free survival (PFS) in CRC [74]. However, this nuclear localization and biological function of DKK1 have not been confirmed in CRC, in other tumors, or in other diseases, and it needs further validation.

In colon cancer, DKK1 functions as an inhibitor of the EMT, and inversely correlates with tumor stage, recurrence, and metastasis. Ectopic overexpression of DKK1 has been shown to restore the epithelial phenotype, downregulate EMT drivers, and reduce expression of stem-cell markers such as CD133 and Lgr5, thus impairing cancer cell tumorigenesis, proliferation, and invasion [75]. Furthermore, homeoboxC6 (HOXC6)-mediated inhibition of DKK1 activates the EMT program, enhancing metastatic dissemination, and correlating with poor clinical outcomes in right-sided colon cancer [76]. Collectively, these insights suggest that DKK1 may play a significant role in pathogenesis and progression across solid and hematological malignancies.

#### 2.2.2. Clinical Implications of Circulating DKK1

The most serious and debilitating complication in MM patients is osteolytic bone disease. Elevated serum DKK1 levels are associated with poor prognosis across multiple tumors, and correlate with increased bone metastatic potential (Table 1). Consequently, circulating DKK1 in the bloodstream may serve as a tumor-specific sensitive biomarker for early-stage detection and monitoring therapeutic efficacy.

Multiple myeloma patients exhibit significantly aberrant protein glycosylation. As previously noted, high circulating concentrations of DKK1 play a major role in MM by promoting osteolytic lesions [6]. Comparative proteomic analysis of MM plasma samples has identified 58 differentially expressed biomarkers. Among them, Fibulin-1 (FBLN1) and DKK1 were significantly upregulated, supporting their potential use in combination as diagnostic markers for MM. Notably, the assessment of 90 newly diagnosed patients and validation in 70 unrelated disease subjects via ROC analysis underscore the clinical translation potential of these findings [77].

Clinically, serum DKK1 levels can be a potential predictor of metastatic tropism in BC, and positively correlate with adverse OS [78]. Specifically, low circulating DKK1 levels correlate with a significant risk of pulmonary metastasis, whereas high levels are associated with increased risk of bone metastasis. This divergent clinical outcome raises significant considerations regarding the systemic use of Wnt pathway-targeting drugs in patients [49]. To date, this is the only study demonstrating differential organotrophic metastasis based on circulating DKK1 levels in both breast cancer and other malignancies. This discovery significantly advances our understanding of the molecular drivers involved in tissue-specific colonization in BC [79,80].

In cervical cancer patients, elevated plasma DKK1 levels correlate with tumors exceeding four centimeters in diameter, and the presence of lymphatic metastasis [13]. Similarly, ovarian cancer (OC) patients have significantly higher plasma DKK1 levels compared to healthy controls, which correlate with advanced OC, peritoneal spreading of the disease, and poor survival. Interestingly, plasma DKK1 is a good biomarker for predicting high recurrence risk in OC patients harboring wild-type *BRCA1/2* [81]. On the other hand, DKK1 levels in the bloodstream of prostate cancer patients versus benign prostate hyperplasia are not a good prognostic marker of tumor progression. While low plasma DKK1 levels are associated with a better outcome, circulating DKK1 levels in PC patients are extremely variable, with no significant correlation with tumor staging or Gleason score. Therefore, DKK1 is currently not considered as a good circulating prognostic marker for PC [82].

NSCLC patients demonstrate significant enrichment of DKK1 in plasma, which positively correlates with TNM staging, lymph node infiltration, and poor survival [83]. In addition, serum DKK1 levels were significantly associated with NSCLC bone metastasis [84], similarly to PC and MM [49,77].

**Table 1:** **Diagnostic and prognostic utility of serological DKK1 in human malignancies.** Summary of the clinical correlation between DKK1 levels in plasma and overall survival, metastasis, and potential use as a prognostic factor.

Tumor Type	DKK1 Correlation with Survival	Role in Metastasis	Potential Prognostic Factor	References
Multiple myeloma	Yes	Bone	Yes	[6,74]
Breast	Yes	Low DKK1 → lung High DKK1 → bone	Yes	[49,75]
Cervical	Yes	Lymphatic	No	[13]
Ovarian	Yes	Peritoneal	Yes	[76]
Prostate	Yes	Unknown	No	[77]
Lung	Yes	Bone	Yes	[78,81]
Hepatocellular	No	No	Yes, with AFP < 20 ng/mL	[82]
Pancreatic	Yes	No	Yes	[16]
Cholangial	No	No	Yes, if combined with CA 19-9	[16]
Esophageal	Yes	No	Yes	[84,85]

In gastrointestinal oncology, CA19-9 represents the established serological standard for the monitoring of pancreatic cancer and cholangiocarcinoma (CCC) [85]. Given that early diagnosis of pancreatic cancer is critical for improving patient outcome, recent findings demonstrate that serum DKK1 levels are significantly high in these patients. Measurement of serological DKK1 protein levels provides a valid diagnostic tool for detecting early-stage pancreatic adenocarcinoma, with more sensitivity than CA19-9, as demonstrated by ROC curve analysis (AUC: 0.919 vs. 0.853) [15]. Additionally, serum DKK1 alone has limited diagnostic and prognostic value in intrahepatic cholangiocarcinoma (ICC), but when combined with CA 19-9 it significantly improves diagnostic sensitivity, particularly by identifying up to 50% of ICC cases that are CA 19-9-negative, and better stratifies survival outcomes [16]. The gold standard for the diagnosis of esophageal squamous cell carcinoma (ESCC) is upper gastrointestinal endoscopy, and so far, no assessment of circulating prognostic factors has been included in ESCC diagnostic protocols. In this regard, plasma DKK1 levels are upregulated in ESCC patients, and measurement of both circulating DKK1 and DKK1 autoantibodies in early and late ESCC can significantly discriminate cancer patients from normal controls. In addition, a significant decrease in DKK1 serum levels is observed in ESCC patients after tumor resection or neoadjuvant treatment, supporting the potential of circulating DKK1 as a reliable biomarker for post-therapeutic monitoring [86,87]. In the context of hepatocellular carcinoma (HCC) detection, while Alpha-Fetoprotein (AFP) remains the most important and widely used prognostic circulating factor, DKK1 significantly improves diagnostic accuracy in patients with AFP levels lower than 20 ng/mL [88].

Collectively, these findings underline the context-dependent upregulation of circulating DKK1 as a potential novel biomarker associated with tumor aggressiveness, metastatic programs, and poor survival across multiple cancers. In conclusion, its greatest clinical advantages lie in early detection, risk stratification, and therapeutic monitoring when used alone or in combination with tumor-specific markers such as CA19-9 or AFP.

### 2.3. DKK1 Orchestrates Immunosuppressive Reprogramming of the TME and Drives Resistance to Immunotherapies

**Figure 4:**
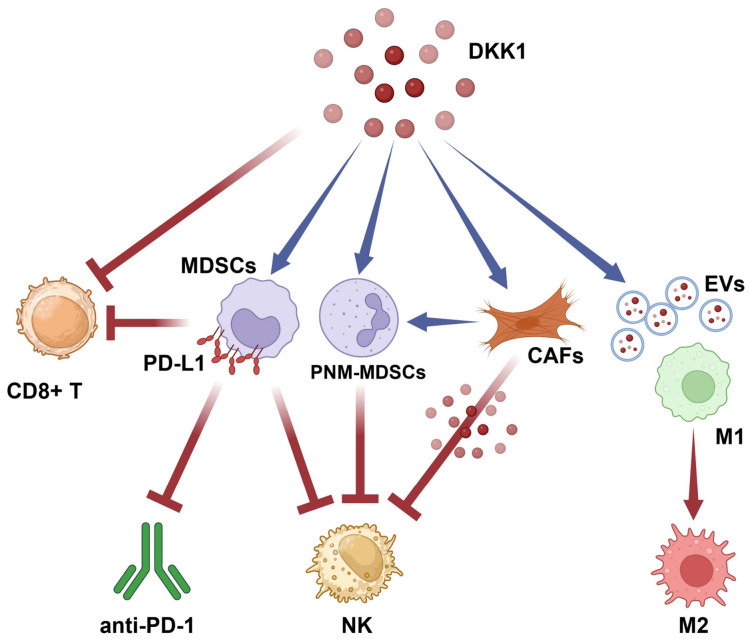
**The comprehensive role of DKK1 in TME reprogramming.** DKK1 acts as a central regulator of immune evasion and stromal remodeling within the TME, facilitating a pro-tumorigenic niche. Abbreviations: CD8^+^ T: cytotoxic T lymphocyte, NK: natural killer, MDSC: myeloid-derived suppressor cell, PNM-MDSC: polymorphonuclear myeloid-derived suppressor cell, CAFs: cancer-associated fibroblasts, EVs: extracellular vesicles, M1: pro-inflammatory macrophage, M2: immunosuppressive macrophage, PD-1: programmed cell death protein 1, PD-L1: programmed death-ligand 1. Figure created in part with BioRender.com (https://app.biorender.com/illustrations/6993ea42467146611ba9bd6a, accessed on 16 April 2026).

Aberrant activation of both canonical and non-canonical Wnt signaling pathways represents a critical mechanism for modulating immune cell proliferation and activation within the tumor niche. Across multiple studies, hyperactivation of tumor-intrinsic Wnt/β-catenin signaling has been identified as a pivotal mediator of immune evasion by inducing an “immune-excluded” phenotype in the tumor microenvironment (TME). This pathway effectively subverts immunosurveillance by suppressing the recruitment of CD103^+^ dendritic cells (DCs) and CD8^+^ effector T cells, thereby preventing the spontaneous priming of antitumor immunity. Beyond these populations, Wnt signaling influences the myeloid compartment by fostering an immunosuppressive and pro-tumorigenic environment. This is characterized by the recruitment of immunosuppressive myeloid-derived suppressor cells (MDSCs) that facilitate metastasis and angiogenesis. Furthermore, elevated WNT signaling frequently correlates with enhanced infiltration of immunosuppressive regulatory T cells (Tregs). In addition, Wnt signaling functions as a central paracrine communication system utilized by stromal cells, particularly cancer-associated fibroblasts (CAFs) and endothelial cells, to promote tumor progression by remodeling the extracellular matrix and driving angiogenesis to reinforce tumor-supportive niches [89,90,91,92]. Consequently, in TMEs characterized by high DKK1 expression, the inhibition of canonical Wnt signaling likely serves to reprogram specific immune and stromal cell populations, thereby impacting tumor progression and immune evasion (Figure 4). This mechanism of immunoregulation is largely conserved across various tumor types.

#### 2.3.1. DKK1 Reprograms the TME to Drive Immune Evasion and Immunotherapy Resistance

In head and neck squamous cell carcinoma (HNSCC), bioinformatic analysis of clinical data indicates that high levels of DKK1 are associated with a more immunosuppressive TME. This includes a correlation with increased angiogenesis, increased infiltration of MDSCs, and upregulation of immunosuppressive cytokines such as TGFB1, TGFB2, and ARG2. These factors, together with the downregulation of T-cell inflammatory gene signatures and reduced TCR diversity, are associated with a limited cytotoxic CD8^+^ T cell antitumor response. Thereby, high DKK1 expression in HNSCC patients is suggested to be a potential predictive biomarker for immune checkpoint inhibition failure [8].

In HCC, preclinical studies using HCC cell lines have demonstrated that DKK1 upregulates the expression of the pro-angiogenic factor VEGFR and the immune checkpoint ligand PD-L1. This process is regulated by the CKAP4/Akt signaling pathway in cancer cells, which subsequently reduces infiltration and cytotoxic activation of CD8^+^ T lymphocytes in vivo [93]. Similar mechanisms of DKK1-mediated immunosuppression occur in melanoma, where DKK1 promotes the trafficking and activation of immunosuppressive MDSCs via β-catenin silencing within the TME [94]. While DKK1’s role in GC tumor proliferation and chemoresistance remains controversial [64,65,67,69,95], its effect on immunosuppression is consistent with findings in other solid tumors, and it remains a consistent driver of poor outcomes. In GC patients, DKK1 expression modulates MDSC recruitment, which in turn contributes to affecting natural killer (NK) and CD8^+^ T cell activation, directly impeding the efficacy of anti-PD-1 immunotherapy [95].

While MDSC recruitment and activation mediated by intratumoral DKK1 in the extracellular space are well characterized, data on mechanisms of CD8^+^ T cell inactivation remain limited. In a preclinical model of OC, ID8 cancer cells overexpressing DKK1 significantly reduced the number of interferon-γ (IFNy)-activated CD8^+^ T cells in the peritoneum of tumor animals compared to those engrafted with ID8 wild-type cells. In addition, lower DKK1 expression in ID8 cancer cells did not translate into a survival benefit in the syngeneic mouse model, highlighting a potential limitation of targeting DKK1 alone as a therapeutic strategy in specific tumors [96]. Sui Q. et al. demonstrated in mismatch repair-deficient CRC that DKK1-mediated CD8^+^ T-lymphocyte inactivation involves the dephosphorylation and nuclear translocation of GSK3β, leading to the downregulation of T-bet, a transcription factor essential for CD8^+^ T cell cytotoxicity. These findings were confirmed in patients where PD-1 resistance was associated with elevated DKK1 levels [97]. Beyond T lymphocytes, NK effector cells are primary targets of DKK1-mediated inhibition in solid tumors. In patients with metastatic castration-resistant prostate cancer, high levels of DKK1 correlate with a significantly higher infiltration of dormant NK cells and lower infiltration of activated NK cells. Preclinical in vivo models show that DKK1 participates in immune evasion by interfering with Wnt-dependent NK cell recruitment and indirectly decreasing NK-cell-activating ligands. Beyond the increased number of quiescent NK cells, the tumor is also infiltrated by an increased number of immunosuppressive M2-like myeloid cells, and lower trafficking of cytotoxic T cells. Interestingly, in this tumor model, the therapeutic benefit of targeting DKK1 requires the presence of NK cells. In fact, the antitumor effect of neutralizing anti-DKK1 antibodies is abrogated upon NK cell depletion [51]. An alternative mechanism of NK cell inactivation was discovered in an immunocompetent breast cancer model, where tumor-cell autocrine DKK1 initiates immune evasion in latency competent cancer (LCC) cells by triggering a quiescent state that masks them from NK-mediated surveillance, and inducing downregulation of NK-activating ligands. Silencing DKK1 in LLC cells exposes them to NK-mediated killing [43]. In the context of breast cancer lung metastases, DKK1 overexpression is associated with increased infiltration of CD11b^+^F4/80^+^ macrophages and CD11b^+^Gr-1^+^Ly6G^+^Ly6C^m^ polymorphonuclear immunosuppressive neutrophils (PNM-MDSCs). Moreover, the DKK1-mediated inhibition of the Wnt/PCP-JNK signaling pathway and PTGS2 expression disrupts immune cell trafficking [49]. Of particular interest is the effect of lung cancer-derived extracellular vesicles in the brain of a lung metastatic murine model. Based on the discoveries of Gan, D.K. et al., circulating extracellular vesicles (EVs) released by lung cancer cells can infiltrate the brain and induce DKK1 upregulation in brain endothelial cells. This paracrine DKK1 secretion into the extracellular matrix induces microglial inactivation and reprograms pro-inflammatory M1-like microglia toward an M2-immunosuppressive state via activation of AMPK signaling in the brain pre-metastatic niche [98].

#### 2.3.2. DKK1 in Cancer-Associated Fibroblasts (CAFs)

CAFs overexpressing DKK1 are emerging as novel critical mediators of immunosuppression and immune evasion in solid tumors. In CRC, fibroblasts represent the predominant cellular source of DKK1. Immunohistochemistry evaluation of DKK1 secretion into the CRC TME in patients demonstrates that DKK1 is primarily secreted by CAFs in the stroma during early stages (non-advanced adenomas), while remaining absent in the normal epithelium. As tumors progress from non-advanced to advanced adenomas, DKK1 expression shifts from the stroma to the tumor adenoduct [99]. Notably, preclinical results show that DKK1 secretion in CRC is significantly higher and more predominant in fibroblasts treated with chemotherapy (5-FU, oxaliplatin, or SN38). This post-therapeutic upregulation is driven by p53-dependent activation of the ERK pathway within CAFs, a mechanism validated in both patient biopsies and in vivo models.

In addition, DKK1 stimulates CAF proliferation through Akt activation [100]. In lung cancer, DKK1 is involved in malignant cell–fibroblast interactions, inducing CAF-mediated release of pro-inflammatory cytokines IL6 and CCL2. The activation of inflammatory CAFs (iCAFs) mediated by cancer-derived DKK1 in lung cancer cell lines in vitro is regulated by the JNK/c-Jun signaling pathway, rather than the CKAP4/Akt pathway [58]. A critical advancement in the field is the recent discovery of an immunosuppressive mechanism driven by myofibroblasts (myCAFs) in human breast cancer. In this model, the extracellular DKK1 is accumulated within the stromal compartment and secreted exclusively by CAFs. The intratumoral accumulation of CAF-derived DKK1 correlates with metastatic progression and reduced NK antitumoral activity in cancer patients. Mechanistically, CAF-derived DKK1 directly interacts with NK cells, abrogating their cytolytic function. This interaction specifically reduces the phosphorylation of AKT, ERK1/2, and S6, while simultaneously downregulating Pfr1, cytokine receptors, and pro-activating receptors such as NKG2D, NKp30, and NKp46. Pharmacologic neutralization of DKK1 with the monoclonal antibody mDKN-01 reverses immunosuppression in vivo, thereby increasing CD8^+^ T cells, NK cells and F4/80^+^ myeloid cells and slowing tumor progression by restoring NK cell function. These findings identify DKK1 as a significant barrier to antitumor immunity in progressive breast cancer [101], thereby providing a strong rationale for the development of novel therapeutic strategies combining DKK1 inhibition with NK-based cellular therapies, such as CAR-NK cells, to overcome the immunosuppressive influence of myCAFs.

Taken together, these studies suggest that DKK1 orchestrates different immunosuppressive programs by regulating the released pro-angiogenic factors, modulating immune checkpoint expression, and reprogramming the trafficking of specific immune cell populations within the TME. These DKK1-mediated mechanisms involve both immune and stromal compartments of the TME, driving immune evasion and resistance to immunotherapies.

### 2.4. DKK1 Therapeutic Inhibition and Clinical Trials

Understanding the pathological transition of DKK1 from a developmental regulator to a facilitator of immune “cold” tumors is essential for the design of modern therapeutic strategies. Recent mechanistic insights have made a significant step forward on elucidating the multifaceted roles of Dickkopf-1 in driving tumor progression, metastatic dissemination, and immune evasion. However, the transition of these fundamental discoveries into clinically approved therapeutic applications remains substantially scarce. MM represented the initial clinical model in which the DKK1 role in tumor progression and metastatic invasion was addressed [6]. Consequently, MM has served as primary cancer model for testing different immunotherapeutic approaches aimed at targeting DKK1. Despite the strong correlation between elevated DKK1 expression and diminished overall survival across several malignancies, the preclinical evaluation of targeted strategies and clinical trials designed to neutralize DKK1 has, to date, not yet reflected the potential suggested by preclinical discoveries on the topic. Over the last decade, novel therapeutic concepts have been designed to modulate the antitumor immune response or to systemically inactivate DKK1, thereby disrupting its downstream oncogenic signaling cascades.

#### 2.4.1. Preclinical Assessment of Immunomodulatory Therapies

DKK1 is highly expressed in MM cancer cells, and HSP70 is a molecular chaperone known to enhance antigenic presentation in immune cells. A fusion DKK1-HSP70 DNA vaccine was developed to promote immunomodulation and antitumor immune response. In MM murine models, the use of the DNA vaccine demonstrated minimal toxicity, increased activation, and proliferation of CD4^+^ and CD8^+^ T cells, and a concomitant reduced number of immunosuppressive FOXP3^+^ regulatory T cells (Tregs) in the spleen. Likewise, post-immunization activated CD8^+^ T cells demonstrated enhanced killing of MM cells in vitro. As a consequence of the immunoregulation post-DKK1-HSP70 vaccination, a significant reduction in tumor burden and prolonged OS was also reported in MM in vivo [102].

Alternatively, a long peptide DKK1 (DKK1-LP) vaccine was evaluated to restore immune response against multiple myeloma in preclinical studies. The 74-amino-acid DKK1 fragment was designed to bind both MHC class I and II molecules in CD4^+^ and CD8^+^ T cells. Interestingly, while DKK1-LP triggered cytotoxic T lymphocytes (CTLs) and T-helper-specific response against DKK1 in both in vitro and in vivo, the study could not prove a beneficial survival effect of the vaccination in MM mice [103].

The integration of DKK1 inhibitions with chimeric antigen receptor (CAR) T cell therapy has been explored in a preclinical model of GC. The DKK1 antagonist WAY-262611 was shown to trigger a pro-inflammatory immune response in the TME, characterized by higher intratumoral trafficking of activated NK cells and reduced infiltration of immunosuppressive M2-like myeloid cells. Interestingly, in vitro treatment of GC cells with WAY-262611 upregulated the NKG2D activation ligands (NKG2DLs), thus sensitizing the tumor to NKG2D-CAR-T cells. In vivo, the combination of WAY-262611 with NKG2D-CAR-T therapy reversed the suppressive tumor microenvironment. The TME reprogramming was hypothesized to improve CAR-T expansion and persistence, leading to significant inhibition of tumor progression without detectable systemic toxicity. However, no significant improvement in the OS in treated mice was reported [104]. Of note, the use of mDKN01 demonstrated substantial therapeutic efficacy in an immunocompetent and subcutaneous GC model. Intratumoral treatment of GC with mDKN01 fostered a robust infiltration of activated IFNy^+^ CD8^+^ T and IFNy^+^ NK cells, as well as more infiltration of myeloid dendritic cells (DCs) and of pro-inflammatory CD86^+^F4/80^+^ myeloid cells, and lower recruitment of immunosuppressive CD163^+^F4/80^+^ myeloid cells. Therefore, mDKN01 treatment had a considerable impact on the tumor burden, animal survival, and the tumor immune response in both TME and metastasis. Importantly, the therapeutic benefit of mDKN01 was abrogated upon depletion of tumor-associated macrophages (TAMs), suggesting that the therapy might work specifically in macrophage-driven immunosuppressive TME [95].

In CRC, third-generation tyrosine kinase inhibitors (TKIs) have been used to indirectly downregulate DKK1 by targeting the FGR-Akt-SP1-DKK1 axis. FGR inhibition induced DKK1 downregulation, promoting antitumor immune modulation characterized by the activation of cytotoxic CD69^+^ T lymphocytes, and significantly enhancing the therapeutic effect of PD-L1 blockade in immunocompetent animals. Similarly to what was noted for GC, while TKIs caused a significant reduction in the tumor burden, no data on improved survival of the animals was shown [70].

The therapeutic response to DKK1 inhibition appears highly tumor-specific. In syngeneic ovarian cancer models, mDKN-01 treatment failed to achieve significant immune reprogramming or survival benefits, highlighting a lack of efficacy in the ovarian murine model [96]. Conversely, mDKN01 demonstrated its therapeutic potential in delaying tumor progression in a B16 melanoma model in vivo. Interestingly, the therapeutic effect was abrogated upon NK cell depletion. Successful DKK1 neutralization was associated with higher tumor infiltration of activated GZMB^+^ NK cells, significant reduction in MDSC infiltration, increased levels of IL15 and IL33 (NK cell-activating cytokines), and reduced levels of the immunosuppressive cytokine TGFβ1. Additionally, the combination of mDKN01 with anti-PD-1 demonstrated synergistic potential in controlling tumor burden compared with monotherapy [105].

#### 2.4.2. Preclinical Testing of Anti-Neoplastic Therapies

Next-generation cytotoxic therapies have been designed to induce targeted cytotoxicity by selectively binding specific MHC class I and II epitopes expressed by cancer cells. The DKK1-A2 monoclonal antibody (mDKK1-A2) was developed to bind surface DKK1-A2 (HLA-A2) complex expressed in MM and in other malignancies. Preclinical studies demonstrate that mDKK1-A2 binds MM cells with high affinity both in vitro and in vivo, subsequently triggering apoptosis-mediated cellular killing. In vivo, this therapeutic effect is characterized by a significant reduction in tumor burden and a remarkable improvement in animal survival. Importantly, the therapeutic efficacy of mDKK1-A2 in vivo was confirmed in murine xenograft models of human mantle cell lymphoma, leukemia, breast cancer, and pancreatic ductal adenocarcinoma. Importantly, the agent was non-toxic and well tolerated [106]. Of note, the therapeutic effect in vivo is impaired by NK cell depletion, consistent with findings regarding the scarce immunomodulatory effects of mDKN01 in the absence of NK cells in vivo [105]. Leveraging the same therapeutic target, Zhang et al. recently designed a promising cellular immunotherapy targeting the DKK1-A2 complex for the treatment of MM. The investigators demonstrated that DKK1-A2 was expressed up to 70 times higher in patient-derived MM cancer cells compared to normal donors. Based on this finding, they engineered chimeric antigen receptor (CAR) T cells to specifically recognize cancer cells expressing the DKK1-A2 complex (DKK1-A2 CAR-T cells). The cytotoxic activity against MM cells was validated in vitro and in vivo, in both immunosuppressed and immunocompetent xenograft models. Interestingly, reduced tumor burden and drastically improved animal survival were confirmed in lung, pancreatic and triple-negative breast cancer mouse models. Furthermore, the DKK1-A2 CAR-T therapy demonstrated long-term durability, effectively neutralizing a second wave of cancer cells injected four weeks post-treatment in vivo. Importantly, the therapy demonstrated target specificity and safety in several preclinical models, with off-tumor toxicity assessments showing no detectable toxicities or damage to normal tissues in either human DKK1 or HLA-A2-transgenic mice [107]. Novel therapeutic strategies also focus on mitigating the development of bone lesions, which represent a debilitating complication in MM patients. A combinatorial therapy of monoclonal antibodies, anti-DKK1 and anti-LRP6, has shown significant induction of bone formation in myeloma-bearing mice, and superior protection against formation of vertebral fractures, when compared to monotherapy. Interestingly, the mechanism of the therapy consists in promoting bone formation by anti-LRP6, while blocking osteolytic processes by neutralizing circulating DKK1 with anti-DKK1. Although the authors state that Wnt stimulation with the DKK1 inhibitor did not promote tumor progression, they did not evaluate the potential risks associated with systemic hyperactivation of Wnt signaling by DKK1/LRP6 dual targeting [108].

Targeting DKK1 in osteosarcoma to reduce bone metastasis and tumor progression has been tested using two different therapeutic strategies. First, in vivo treatment of osteosarcoma xenograft mice with anti-DKK1 monoclonal antibody BHQ880 was able to sequester DKK1 from the bloodstream, resulting in improved bone differentiation, reduced metastasis, and increased animal survival [109]. The second strategy aimed to block DKK1 transcription using a vivo-morpholino (DKKMo, anti-human DKK-1 mRNA) against osteosarcomas both in vitro and in vivo. The DKK1Mo-mediated DKK1 knockdown in osteosarcoma in vivo stimulated tumor necrosis by blocking Aldh1a1, reduced osteolytic bone damage by inhibiting DKK1-mediated suppression of osteoblastogenesis, and sensitized cancer cells to doxorubicin without inducing systemic toxicity [110].

In CRC, DKK1 is upregulated in tumor-bearing animals resistant to the chemotherapeutic agent oxaliplatin, where the drug-resistance mechanism is regulated by the DKK1-CKAP4 axis. DKK1-CKAP4 binding occurs though the CDR1 protein domain in DKK1 and the LZ protein domain in CKAP4. The LZ protein domain binds with high affinity to both exogenous and endogenous DKK1 protein. The treatment of oxaliplatin-resistant CRC tumors in vivo with the recombinant LZ protein domain suppressed cancer cell proliferation and stimulated apoptosis, effectively inhibiting tumor growth [33].

As previously reported in this review, upregulation of the EGFR pathway post-chemotherapy induces the overexpression of DKK1 in breast cancer cells and in peripheral neurons [50]. In these tumor-bearing mice, cotreatment with paclitaxel and anti-DKK1 enhanced therapeutic efficacy in reducing tumor growth and increasing tumor infiltration of activated GZMB^+^CD8^+^ T cells. Of note, anti-DKK1 therapy ameliorated neuropathic pain and peripheral nerve injury, which are common adverse effects of paclitaxel treatment [50].

Finally, direct inhibition of CKAP4 can be adopted to indirectly block DKK1-driven cancer cell proliferation. The humanized anti-CKAP4 antibody Hv1Lt1 acts as a DKK1 antagonist for the CKAP4 receptor. The use of Hv1Lt1 in subcutaneous pancreatic murine models demonstrates significantly reduced tumor growth in both immunocompromised and immunocompetent murine strains. In addition, the treatment promoted increased trafficking and tumor infiltration of cytotoxic CD8^+^ T cells.

#### 2.4.3. Clinical Evaluation of DKK1 Target Therapies

DKN-01, a humanized monoclonal antibody targeting DKK1, has emerged as a promising therapeutic agent across multiple solid tumor types. Clinical development has focused primarily on gastroesophageal adenocarcinomas. In a Phase Ib trial (NCT02013154) combining DKN-01 with pembrolizumab, anti-PD-1/PD-L1-naïve patients with DKK1-high gastroesophageal tumors had an objective response rate (ORR) of 50% versus 0% in DKK1-low patients. The association between DKK1 expression and progression-free survival (PFS) was independent of PD-L1 status [111]. In the Phase II DisTinGuish trial (NCT04363801), DKN-01 combined with tislelizumab and fluoropyrimidine/oxaliplatin chemotherapy demonstrated a 73% ORR in the first-line setting, with a 90% ORR in DKK1-high tumors [112]. In the second-line setting, DKN-01 plus tislelizumab achieved a 21.7% ORR in DKK1-high gastroesophageal cancers, comparing favorably to historical benchmarks (NCT04363801) [113].

Beyond gastroesophageal cancers, DKN-01 has demonstrated activity across multiple malignancies. In recurrent endometrial carcinoma, DKN-01 monotherapy achieved a 25% ORR in DKK1-high tumors, with a median PFS of 4.3 months and overall survival (OS) of 11.0 months. Comparatively, DKK1-low tumors had a 0% ORR, PFS of 1.8 months, and OS of 8.2 months (NCT03395080) [114]. In metastatic, castration-resistant prostate cancer, DKN-01 combined with docetaxel produced partial responses in 5/7 (71%) of evaluable patients, and this activity was not DKK1-level-dependent (NCT03837353) [115]. The DeFianCe trial (NCT05480306) in microsatellite stable colorectal cancer showed a 30% ORR and 93% disease control rate when DKN-01 was added to chemotherapy and bevacizumab in the second-line setting [116]. In biliary tract cancer, DKN-01 combined with gemcitabine/cisplatin was well tolerated but did not exceed the historical efficacy of chemotherapy alone, though biomarker data suggested potential antiangiogenic and immunomodulatory effects (NCT02375880) [117].

Across all trials, DKN-01 demonstrated excellent tolerability with no dose-limiting toxicities. Adverse events in these trials are primarily attributable to combination chemotherapy rather than the antibody itself [111,112,113,115,117]. The finding that DKK1 expression serves as a predictive biomarker in gastroesophageal and endometrial cancers, but not in prostate cancer, suggests that patient selection strategies may need to be tumor-specific and warrants further investigation in ongoing randomized trials.

## 3. Conclusions and Future Perspectives

The findings presented in this review underscore a longstanding dichotomy in the DKK1 literature—how the same molecule can appear tumor-suppressive or tumor-promoting depending on epigenetic state, targeted pathway, and treatment context. While DKK1 was historically classified as a Wnt antagonist, it is frequently overexpressed in advanced malignancies, functioning as a signaling hub that facilitates tumor malignancy through the activation of the CKAP4/PI3K/Akt pathway. This functional dichotomy becomes particularly evident during tumor progression, as DKK1 facilitates immunosuppressive tumor conditions, mechanisms of chemotherapy and immunotherapy resistance, immune reprogramming of the tumor microenvironment, and metastasis. However, the effect of DKK1 is tumor-specific: there are tumors that benefit from higher levels of DKK1, and tumors that require DKK1 silencing to maintain active tumorigenesis. DKK1 gene expression is governed by a complex array of epigenetic regulatory mechanisms, including induction by p53 in response to DNA damage, PRMT5 activation, or a negative feedback loop with the Wnt pathway via TCF/LEF transcription factors. Conversely, expression is often modulated by epigenetic silencing through promoter hypermethylation or non-coding RNAs, such as miR-493 and miR-501-5p, which drive aberrant Wnt activation and cancer stemness. In the context of metastasis and chemoresistance, DKK1 promotes epithelial-mesenchymal transition (EMT) and vasculogenic mimicry, particularly in lung and breast cancers, while facilitating resistance to chemotherapies.

Despite these advancements, several critical gaps remain in our understanding of the factors governing DKK1 expression. Specifically, it is not yet clear which secreted factors or other intratumoral stimuli may contribute to the epigenetic modulation of the DKK1 functional paradox within the tumor niche. While p38 MAPK and hypoxia have been identified as upstream regulators, the broader landscape of external cues that trigger these epigenetic shifts requires further investigation. Furthermore, while this review has highlighted that DKK1 secretion has been mediated by cancer cells and cancer-associated fibroblasts, it is still unclear which other cell types within the TME, such as endothelial or specific immune cell populations, could secrete DKK1 to modulate the local environment.

A similar ambiguity exists regarding the origins of systemic DKK1. So far, we do not know if high circulating levels of DKK1 are derived from cancer cell secretion, or if there are other cell types involved in contributing to the protein’s presence in the bloodstream. Identifying these sources is essential for refining DKK1 as a diagnostic and surveillance biomarker across different cancer types. Moreover, the discovery of a non-canonical nuclear localization of DKK1 in colorectal cancer suggests a functional versatility that has yet to be fully validated. Therefore, there is a clear highlighted need to identify if other signaling pathways may be activated or blocked by DKK1 in tumors beyond the traditional Wnt and CKAP4 cascades. Transitioning these fundamental discoveries into clinically approved therapies will require a deeper understanding of how DKK1 coordinates its functional dichotomy across tissues, disease stages, and cellular compartments. Finally, several tumors, including thyroid and stomach carcinoma, exhibit significantly high DKK1 expression levels. However, in these tumors the role of DKK1 remains to be fully investigated (Figure 2). Moving forward, future work should focus on several priority areas for advancing the translational relevance of DKK1 biology. First, defining the cellular sources and tissue origins of circulating DKK1 will be essential to clarify its value as a biomarker. Second, the biological relevance of nuclear DKK1 localization requires further validation, including its molecular interactors and downstream effects. Third, additional studies are needed to evaluate whether DKK1 expression or epigenetic status can be used to guide patient stratification and therapeutic decision-making in clinical settings.

## Data Availability

No new data were created or analyzed in this study. Data sharing is not applicable.
